# Determinants of Patients’ Intention to Use Remote Monitoring Service for Cardiac Implantable Electronic Devices: An Extended Technology Acceptance Model Study in Taiwan

**DOI:** 10.3390/healthcare14121802

**Published:** 2026-06-22

**Authors:** Teh-Kuang Sun, Shu-Hui Chuang

**Affiliations:** 1 Department of Business Administration, Asia University, Taichung 41354, Taiwan; 2 Department of Internal Medicine, Chung-Kang Branch, Cheng Ching Hospital, Taichung 40764, Taiwan

**Keywords:** remote monitoring, cardiac implantable electronic devices, technology acceptance model

## Abstract

**Background/Objectives**: Remote monitoring (RM) of cardiac implantable electronic devices (CIEDs) has been associated with potential clinical and economic benefits; however, its adoption among patients remains limited in some healthcare settings. This study examined patients’ intention to use RM services by applying an extended Technology Acceptance Model (TAM) that incorporates perceived effectiveness (PE), perceived barriers (PB), perceived threat (PT), and economic considerations, as well as the influence of socioeconomic factors. **Methods**: A cross-sectional survey was conducted among 104 patients with CIEDs in Taiwan using validated questionnaires. Structural equation modeling (SEM) was employed to examine the relationships among the proposed constructs. The association between intention to use and actual service utilization was explored. The correlations between sociodemographic factors and the constructs were analyzed using analysis of variance (ANOVA). **Results**: SEM showed that perceived effectiveness (PE), perceived usefulness (PU) and perceived ease of use (PEOU) were significantly associated with intention to use RM services, with economic considerations also having a significant contribution. Intention to use RM services further predicted actual adoption. However, PB and PT did not moderate these relationships. Sociodemographic factors influenced RM acceptance, with younger, more educated, employed, higher-income, and professionally employed patients reporting stronger perceptions and greater intention to use RM. **Conclusions**: This study reinforces the TAM framework in the context of health-related technology adoption. Overall, the adoption of RM services is complex and shaped by psychological, economic, and demographic factors, highlighting the need for user-friendly design, targeted education on clinical benefits, and flexible pricing and reimbursement strategies to improve equitable and sustained use.

## 1. Introduction

Since the invention of the cardiac pacemaker in 1952, cardiac implantable electronic devices (CIEDs) like pacemakers and implantable cardioverter-defibrillators have become essential in managing arrhythmias and heart failure [1,2,3]. Traditionally, patients with CIEDs require regular in-clinic visits, which can burden both patients and healthcare systems, especially for those with limited mobility, those living in remote areas, or those requiring long-term monitoring [4].

Remote monitoring (RM) enables clinicians to remotely assess device status and patient conditions [5], allowing early detection of malfunctions or clinical events, improving safety, and reducing unnecessary hospital visits [6]. Evidence suggests RM enhances disease management, healthcare efficiency, and reduces utilization [7,8]. Despite these potential advantages, however, in Taiwan, adoption remains limited; fewer than 0.5% of CIED patients use RM [9]. Understanding factors influencing patient acceptance is essential to improve implementation.

The Technology Acceptance Model (TAM) has been widely used to explain technology adoption in healthcare [10,11], proposing that behavioral intention is influenced by perceived usefulness (PU) and perceived ease of use (PEOU) [12]. Although TAM has strong explanatory power, additional factors may affect acceptance of digital health technologies [10,13]. In the context of RM, patients may also consider perceived effectiveness, economic costs, follow-up barriers, and perceived disease threat when deciding whether to use the technology [14,15,16].

This study extends the traditional TAM by incorporating factors relevant to RM adoption. It includes perceived effectiveness (PE), reflecting patients’ belief in RM’s ability to improve health management [14], and economic considerations regarding its financial impact [16]. It also examines perceived barriers (PB) (e.g., time, transportation, and lack of support) [15] and perceived threat (PT), defined as perceived vulnerability to worsening cardiac conditions without regular follow-up [17]. In addition, this study examines the relationship between behavioral intention and actual use, as well as the effects of socio-demographic characteristics on RM adoption.

## 2. Theoretical Background and Hypothesis Development

### 2.1. Remote Monitoring Services of Cardiac Implantable Electronic Devices

CIEDs consist of a pulse generator and leads that deliver electrical impulses and sense cardiac activity [18]. They are indicated for bradyarrhythmias, heart failure, and life-threatening ventricular arrhythmias. Patients require regular follow-up, typically every six months, which may impose significant financial and time burdens on patients, caregivers, and healthcare systems [7]. To address these challenges, RM has emerged as a technological solution. This development has been described as “a paradigm shift in implantable device technology” [19].

RM of CIEDs is clinically feasible and enables earlier detection of arrhythmias and device malfunctions. Compared with routine follow-up, RM reduces hospitalizations, atrial arrhythmias and stroke-related events, and unscheduled clinic visits, thereby improving care quality and patient outcomes [5,20,21]. RM is also cost-effective, enabling earlier intervention, reducing hospitalizations, mortality risk, and outpatient visits, and resulting in significant cost savings compared with conventional follow-up [7,8,20]. Given its well-established clinical utility, since 2015, the Heart Rhythm Society has recommended remote monitoring as a standard tool to monitor clinical and device-related events in patients with CIEDs [22].

### 2.2. Technology Acceptance Model

Introduced by Fred D. Davis in the late 1980s, TAM is a widely validated framework for explaining and predicting user acceptance of information technology [12,23]. Due to its robustness and parsimony, it has been widely applied and extended across diverse technological contexts [13]. TAM proposes that behavioral intention to use a system is primarily determined by two beliefs: PEOU and PU. PEOU refers to the degree to which a user believes that using a system requires minimal effort, encompassing factors such as clarity, learnability, intuitiveness, and cognitive demand. PEOU influences both PU and behavioral intention to use the system. PU refers to the degree to which a person believes that using a particular system or technology will enhance performance or help achieve desired outcomes [12,23]. In RM settings, PEOU is determined by the extent to which patients find the RM ser-vice easy to learn and operate, as well as by the clarity of its interface and the effort required for its use. PU refers to the degree to which the RM service contributes to im-proved clinical outcomes by enabling the early detection of cardiac events, improving patients’ quality of life, and enhancing communication with healthcare providers. When PEOU and PU align with users’ expectations, they positively influence behavioral intention and actual technology use.

In the healthcare domain, TAM-based studies have primarily focused on technology adoption among healthcare providers, particularly in areas such as electronic health records, telehealth systems, and mobile health applications, rather than on the patient’s perspective, studies adopting a patient-centered TAM perspective remain limited [10,11,24]. Although TAM has been applied in the context of human implantable technologies, it has not yet been extended to research on the remote monitoring of CIEDs [25].

Regular follow-up of CIEDs is essential for assessing device status and detecting abnormalities such as battery depletion, threshold changes, or inappropriate shocks, thereby ensuring treatment quality and patient safety. In this context, patients’ PE of remote monitoring may influence their acceptance of the technology. By definition, efficiency refers to the ease with which users can access information provided by the technology, whereas effectiveness pertains to the quality and usefulness of that information [14]. PE refers to user’s evaluation of a system’s ability to achieve intended goals, including its functionality, accuracy, and efficiency. It reflects whether patients believe the system can support health management effectively [26]. When patients perceive RM services as effectively tracks CIEDs status, detecting abnormalities early, and enabling timely intervention, they are more likely to view it as beneficial, thereby increasing their intention to adopt and continue using the service [27].

Drawing on the TAM framework, these factors provide a basis for understanding patients’ intention to use RM services. PEOU reflects willingness to use RM after learning that it is easy to operate; when patients find the system simple to understand and use, the cognitive burden is reduced, thereby increasing confidence and intention to adopt RM. PU reflects willingness after understanding what RM is and its benefits; when patients recognize its value in continuous monitoring and early detection of cardiac abnormalities, their motivation to use RM increases. PE reflects patients’ belief that RM can effectively achieve clinical goals such as accurate device tracking and timely intervention. When patients perceive RM as effective, their acceptance and sustained intention to use the service are strengthened.

Based on these evidences, we hypothesized the following:

**H1:**  *PEOU of the RM services positively affects patients’ behavioral intention to use the RM services.*

**H2:**  *PU of the RM services positively affects patients’ behavioral intention to use the RM services.*

**H3:**  *Behavioral intention to use the RM services positively affects actual usage behavior.*

**H4:**  *PEOU of the RM services positively affects PU.*

**H5:**  *PE of the RM services positively affects patients’ behavioral intention to use the RM services.*

**H6:**  *PE of the RM services positively affects PEOU.*

**H7:**  *PE of the RM services positively affects PU*.

### 2.3. Moderating Variables

PB and PT are key constructs derived from the HBM, developed in the 1950s to understand individuals’ engagement in health screening or disease prevention [28,29,30]. The model assumes that a person’s willingness to engage in health behaviors is primarily influenced by their personal beliefs about the health issue and available preventive strategies. The integration of HBM constructs into the TAM has proven theoretically feasible for e-health studies [31].

PB refers to an individual’s assessment of obstacles that may hinder adopting recommended health actions, which could be physical, psychological, logistical, or social [31]. For patients with CIEDs, PB may arise from issues such as transportation difficulties, time constraints, lack of support, or psychological reluctance toward follow-up care, all of which can impede consistent healthcare engagement [15].

PT refers to the belief in one’s vulnerability to a health risk and the perceived seriousness of its consequences [27,28,29]. CIED patients may feel increased vulnerability if they fear worsening arrhythmias or heart failure from irregular follow-ups. Perceiving biannual visits as insufficient may heighten health concerns, highlighting the need for timely and consistent monitoring [15,29].

PB and PT have typically been examined as direct predictors or components of interaction models within the Health Belief Model (HBM) rather than as moderators in health behavior research [27,28,29]. However, some evidence suggests that HBM constructs may also function as moderators. A scoping review identified PB as a strong predictor, with potential moderating effects depending on study design and timing of measurements [30]. Similarly, Im et al. found that perceived risk, a component of PT, moderated relationships between behavioral intention and key TAM constructs, helping explain inconsistencies in PU and PEOU [32]. Subsequent studies further indicate that the effects of PB and PT vary across psychological and contextual factors. For example, during the COVID-19 pandemic, perceived disease threat moderated the relationship between behavioral determinants and the use of health prevention applications [33]. These findings provide a theoretical foundation for examining PT and PB as moderators in the relationship between TAM constructs and the intention to use RM. Thus, we hypothesize the following:

**H8:**  *PB enhances the positive relationship between PE and behavioral intention to use.*

**H9:**  *PB enhances the positive relationship between PEOU and behavioral intention to use.*

**H10:**  *PB enhances the positive relationship between PU and behavioral intention to use.*

**H11:**  *PT enhances the positive relationship between PE and behavioral intention to use.*

**H12:**  *PT enhances the positive relationship between PEOU and behavioral intention to use.*

**H13:**  *PT enhances the positive relationship between PU and behavioral intention to use.*

### 2.4. Economic Considerations

Most studies on healthcare economics focus on provider-centered approaches, aiming to reduce costs and improve cost-effectiveness. Evidence shows that RM service for CIEDs reduces mortality and cardiovascular hospitalizations, leading to significant cost savings for healthcare institutions [6,7,8,19,20]. However, there is a research gap in adopting a patient-centered approach to understand CIED patients’ perceptions of RM services pricing and adoption from an economic perspective [15,16].

Patients’ economic considerations strongly influence adoption of healthcare technologies. Even if perceived as necessary, high out-of-pocket costs may exceed patients’ budgets and reduce perceived value, particularly when benefits are not immediately apparent [34].

From a behavioral economics perspective, individuals are more likely to accept pricing they perceive as fair and tailored to their needs. When multiple options are available, patients’ choices are shaped by both financial constraints and personal needs. Preferences for particular pricing structures may also reflect perceived fairness and value alignment [35]. A severity-adjusted pricing model may enhance perceived fairness and affordability, thereby improving acceptance of remote monitoring services. From these suggestion and evidences, thus, we hypothesize the following:

**H14:**  *Economic considerations regarding the use of RM services positively affect behavioral intention to use.*

Furthermore, patients’ decisions to utilize specific healthcare services are mainly influenced by five key factors: sociodemographic characteristics, PT, perceived benefits, PB, and other sources of information [16]. From this perspective, integrating patients’ sociodemographic characteristics to evaluate their relationships with the above-mentioned constructs may provide better insight into the low uptake of RM services among patients with CIED.

**Figure 1:**
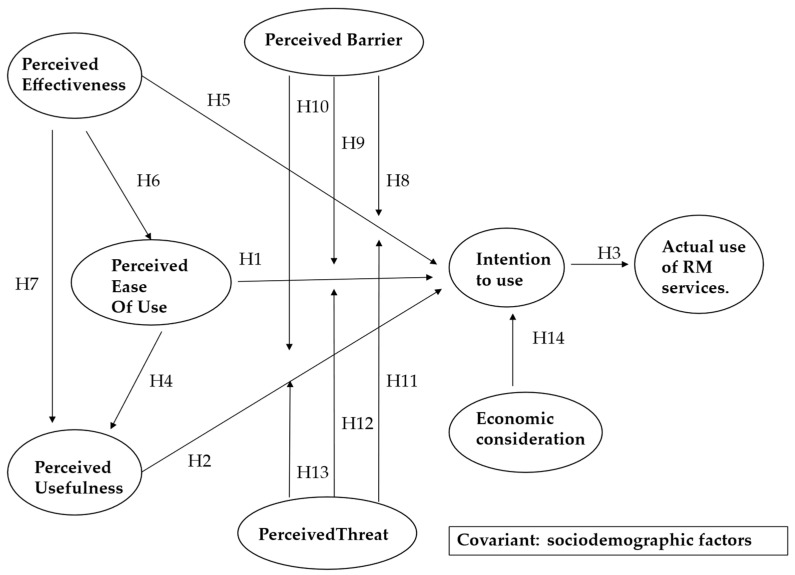
The hypothesized model.

The proposed research model is presented in Figure 1.

## 3. Materials and Methods

### 3.1. Context and Participants

This research was designed as an observational, cross-sectional study to understand of patient’s intention of use and actual use with RM services of CIED in Taiwan between 1 June 2025 and 30 November 2025.

The study followed the Helsinki–Tokyo–Venice guidelines for human research, approved by the Institutional Review Board of Cheng Ching Hospital on 19 April 2025, IRB number HP250009.

A total of 104 patients with CIED were randomly included. Socio-demographic characteristics of the participants are presented in Table 1. Most were aged 65–80 years (40.4%), followed by those aged 45–64 years (27.9%) and over 80 years (26.0%), while only 5.8% were aged 31–44 years. Slightly more than half were male (54.8%). Regarding education, 33.7% had completed high school, 31.7% held a college or university degree, 28.8% had primary education, and 5.8% had graduate-level education. The majority lived with family members (90.4%), while 9.6% lived alone. Nearly half of the participants (49.0%) reported an annual income below 343,080 NTD, whereas only 6.7% exceeded 1,330,001 New Taiwanese Dollar (NTD). Participants represented diverse occupational backgrounds, with freelancers or homemakers comprising the largest group (44.2%), followed by public service (13.5%), agriculture-related sectors (12.5%), and manufacturing or construction (10.6%). Overall, 65.4% were retired. Most participants reported at least one chronic disease (60.6%), while 25.0% had two or more and 14.4% reported none. The primary indications for cardiac implantable devices were sick sinus syndrome or atrioventricular block (80.8%), followed by heart failure (9.6%) and ventricular tachycardia (9.6%). Of the 104 patients included in this study, 12 (11.5%) utilized remote monitoring. The utilization rates were 30.0% (3/10) among CRT recipients, 30.0% (3/10) among ICD recipients, and 7.1% (6/84) among pacemaker recipients.

### 3.2. Measures

The TAM construct of PEOU was assessed using four items (e.g., “It is easy to learn how to use the remote monitoring service.”), PU was measured through six items (e.g., “Using the remote monitoring service helps detect heart abnormalities early.”), PE was measured using six items (e.g., “Using the remote monitoring service helps me keep track of my device.”). PB was assessed using five items (e.g., “I am sometimes unable to attend follow-up visits due to personal reasons”), and PT was measured through five items (e.g., “I would feel uneasy if I did not have regular follow-up for my device”). Economic consideration was assessed through three items (e.g., “The current cost of remote monitoring services is expensive for me.”). Intention to use was measured with three items (e.g., “I am willing to use the remote monitoring service after learning what it is”). All items were measured using a five-point Likert scale ranging from 1 (strongly disagree) to 5 (strongly agree). These items were designed and adapted based on established theoretical frameworks and previous studies. The actual use of the remote monitoring service was assessed using a dichotomous response (“yes” or “no”).

### 3.3. Data Analysis

Sample characteristics and the reliability and validity of the measurement model were assessed using descriptive statistics, Cronbach’s alpha, composite reliability (CR), and average variance extracted (AVE) in SPSS version 25.0 (IBM Corp., Armonk, NY, USA), and Heterotrait–Monotrait (HTMT) ratio was tested in Jamovi version 2.6.26.0 [36].

Confirmatory factor analysis (CFA) was conducted to assess the measurement model prior to structural analysis. Model fit was evaluated using multiple fit indices, including absolute, incremental, and parsimonious fit measures.

Structural equation modeling (SEM) was used to test the hypotheses. Bootstrapping with repeated subsampling assessed the significance of effects. The moderating effects of PT and PB were examined. The association between intention to use and actual use of remote monitoring services was analyzed using binary logistic regression. The correlation between sociodemographic factors and constructs included in the proposed research model is analyzed by Analysis of Variance (ANOVA). All analyses were conducted using Jamovi version 2.6.26.0.

## 4. Results

### 4.1. Reliability and Validity

The measurement model was evaluated for reliability, convergent validity, and collinearity, with all constructs demonstrating satisfactory psychometric properties (Table 2). TAM-related constructs showed strong performance: PE, PEOU, and PU had high factor loadings (≥0.86), excellent reliability (α ≥ 0.95; CR ≥ 0.95), and strong convergent validity (AVE ≥ 0.85). Intention to use also showed high loadings (0.90–0.98), with excellent reliability (α = 0.97; CR = 0.97) and convergent validity (AVE = 0.91). Economic consideration showed more variable loadings (0.43–0.80) but acceptable reliability (α = 0.75; CR = 0.77) and validity (AVE = 0.55). Item E1 (loading = 0.43) was retained as it captures patients’ perceived affordability of remote monitoring services. PB and PT demonstrated adequate loadings (0.62–0.90), good reliability (α = 0.90 and 0.84), and acceptable validity (AVE = 0.66 and 0.54). VIF values ranged from 1.214 to 4.725. Although PU (4.513), PE (4.725) and intention to use (4.133) slightly exceeded the conservative threshold of 3.3, they remained below the acceptable upper limit of 5, indicating no serious multicollinearity.

Discriminant validity was confirmed using the heterotrait–monotrait ratio (HTMT) and the Fornell–Larcker criterion. All HTMT values were below 0.90; although PE–PU (0.904) was near the threshold, indicating substantial conceptual overlap, however, because the two constructs represent distinct but theoretically related aspects of users’ evaluations of RM services, they were retained as separate constructs in the analysis [37]. Additionally, the square root of AVE exceeded inter-construct correlations for all constructs. Overall, results support adequate discriminant validity and indicate no serious multicollinearity concerns (Table 3).

### 4.2. The Results of SEM, Binomial Logistic Regression Analysis, and Moderating Effects

A confirmatory factor analysis (CFA) was conducted to evaluate the measurement model. The model yielded a significant chi-square (χ^2^) statistic (χ^2^(309) = 578, *p* < 0.001), which is expected given its sensitivity to sample size. The chi-square to degrees of freedom ratio (χ^2^/df = 1.87) indicated an acceptable model fit. The standardized root mean square residual (SRMR = 0.063) and root mean square error of approximation (RMSEA = 0.090, 90% confidence interval [CI]: 0.080–0.103) suggested acceptable absolute fit. Incremental fit indices indicated satisfactory fit, including the comparative fit index (CFI = 0.925), Tucker–Lewis index (TLI = 0.914), relative noncentrality index (RNI = 0.925), and incremental fit index (IFI = 0.926), all exceeding the recommended threshold of 0.90. The normed fit index (NFI = 0.851) and relative fit index (RFI = 0.830) were slightly below the recommended cutoff; however, the goodness-of-fit index (GFI = 0.943) and adjusted goodness-of-fit index (AGFI = 0.925) indicated good model fit. Finally, the parsimonious normed fit index (PNFI = 0.744) and parsimony goodness-of-fit index (PGFI = 0.710) suggested adequate model parsimony. Overall, the measurement model demonstrated an acceptable fit.

**Figure 2:**
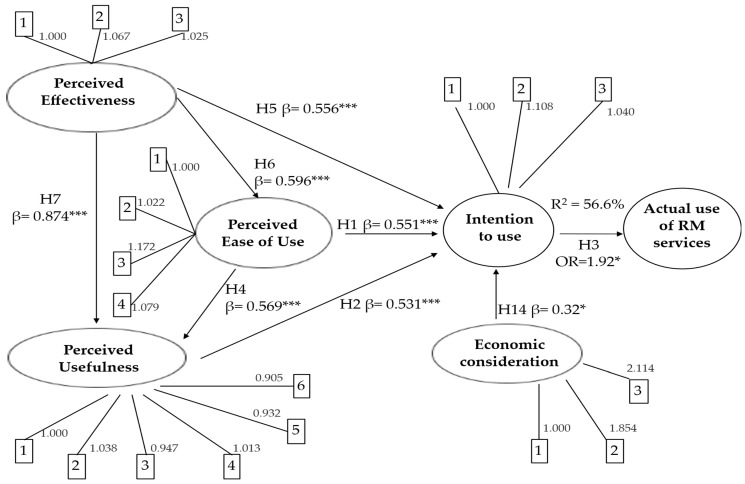
The model results. Ovals indicate latent variables; squares indicate observed variables. Numbers in square indicates the observed variables. * *p* < 0.05, *** *p* < 0.001.

The SEM revealed several significant pathways influencing intention to use RM technology (Figure 2). PEOU positively associated with both intention to use (β = 0.551, *p* < 0.001) and PU (β = 0.569, *p* < 0.001), thereby supporting Hypotheses 1 and 4. PU also exerted linked to greater intention to use (β = 0.531, *p* < 0.001), supporting Hypothesis 2. PE contributed positively on PEOU (β = 0.596, *p* < 0.001), PU (β = 0.874, *p* < 0.001), and intention to use (β = 0.556, *p* < 0.001), thereby supporting Hypotheses 5–7. In addition, economic considerations significantly linked to intention to use (β = 0.032, *p* < 0.05), supporting Hypothesis 14. To determine overall model adequacy, the coefficient of determination (R^2^) was assessed, revealing a value of 56.6% for intention to use. Overall, these findings indicate that PE, PEOU, PU, and economic considerations are significant contributors of intention to use RM service.

A binomial logistic regression analysis was conducted to examine the association between intention to use RM and actual RM use (Figure 2). The overall model was statistically significant (χ^2^ = 9.55, df = 1, *p* < 0.01), indicating that intention to use RM significantly improved the prediction of actual RM use. Model fit was acceptable, as evidenced by the deviance statistic (64.8), AIC (68.8), BIC (74.1), McFadden’s R^2^ (0.128) and the Hosmer–Lemeshow goodness-of-fit test (χ^2^ = 0.068, df = 2, *p* > 0.05), suggesting adequate model calibration. The Nagelkerke R^2^ value was 0.17, indicating that approximately 17% of the variance in actual RM use was explained by intention to use RM. Intention to use RM was a significant predictor of actual RM use (β = 0.651, SE = 0.261, *p* < 0.05). Specifically, each one-unit increase in intention was associated with a 1.92-fold increase in the odds of actual RM use (OR = 1.92, 95% CI: 1.15–3.20). Therefore, Hypothesis 3 was supported.

The moderation effects of PB and PT were analyzed and tested at the factor-score (observed variable) level. Predictor and moderator variables were mean-centered prior to the creation of interaction terms. The interaction terms (PU × PB, PE × PB, PEOU × PB, PU × PT, PE × PT, and PEOU × PT) were entered into the regression models, and their significance was assessed using bootstrap estimation with 5000 resamples. A significant interaction coefficient was interpreted as evidence of moderation. The results showed that PB did not significantly moderate the relationships between PU, PE, PEOU, and intention to use. The interaction effects of PE × PB (β = 0.009, *p* > 0.05), PEOU × PB (β = 0.003, *p* > 0.05), and PU × PB (β = −0.001, *p* > 0.05) were all non-significant. Similarly, PT did not significantly moderate these relationships. The interaction effects of PE × PT (β = −0.037, *p* > 0.05), PEOU × PT (β = 0.001, *p* > 0.05), and PU × PT (β = −0.020, *p* > 0.05) were also non-significant. Although the main effects of PU, PE, and PEOU on intention to use were significant, these relationships remained stable across different levels of PB and PT. Therefore, Hypotheses 8–13 were not supported.

### 4.3. The Effects of Sociodemographic Variables

The effects of sociodemographic variables analyzed by ANOVA, along with the corresponding Tukey’s HSD post hoc comparisons, are shown in Table 4. These results indicated younger patients reported higher levels of PB and PT during CIED management and follow-up (PB: F(3, 100) = 4.71, *p* < 0.01, η^2^p = 0.124; PT: F(3, 100) = 3.30, *p* < 0.05, η^2^p = 0.09), and demonstrated a stronger intention to use remote services (F(3, 100) = 3.42, *p* < 0.05, η^2^p = 0.093) compared to older patients. Higher educational attainment was also associated with greater PB and PT (F(3, 100) = 5.41, *p* < 0.01, η^2^p = 0.133 and F(3, 100) = 5.50, *p* < 0.01, η^2^p = 0.142), a stronger belief in the effectiveness and usefulness of the service (PE: F(3, 100) = 4.77, *p* < 0.01, η^2^p = 0.125; PU: F(3, 100) = 4.55, *p* < 0.01, η^2^p = 0.120), and greater intention to adopt it (F(3, 100) = 6.98, *p* < 0.001, η^2^p = 0.173). Technological professionals showed significantly greater adoption intention than those in freelance or household roles (F(7, 96) = 3.21, *p* < 0.01, η^2^p = 0.19). Employed patients experienced higher PB and PT, perceived the service as more effective, and had stronger intentions to use it (PB: F(1, 102) = 12.62, *p* < 0.01, η^2^p = 0.11; PT: F(1, 102) = 4.54, *p* < 0.05, η^2^p = 0.043; PE: F(1, 102) = 5.01, *p* < 0.05, η^2^p = 0.047; intention to use: F(1, 102) = 7.03, *p* < 0.01, η^2^p = 0.065) compared to retirees. Patients’ annual income was significantly associated with perceptions and intention to use RM services, patients with an income of 590,001–1,330,000 NTD perceived more barriers to CIED in-clinic follow-up and perceived the RM service to be more effective than those with an income of 343,080–590,000 NTD. (F(3, 100) = 3.48, *p* < 0.05, η^2^p = 0.095; F(3, 100) = 4.54, *p* < 0.01, η^2^p = 0.12). For intention to use, participants with higher annual income were associated with a higher intention to use the RM service than those with lower annual income (F(3, 100) = 6.61, *p* < 0.001, η^2^p = 0.165).

## 5. Discussion

Despite the established clinical and economic benefits of remote monitoring and its recommendation by the Heart Rhythm Society since 2015 [22], adoption in Taiwan remains low [9]. This study applied the TAM, incorporating external factors and HBM constructs (PT and PB) as moderators, to investigate willingness to use remote monitoring among Taiwanese patients with CIEDs.

This study has modest sample size (N = 104), includes seven latent constructs and 29 indicators, representing a moderately complex SEM. Nevertheless, the measurement model demonstrated strong psychometric properties, with satisfactory standardized factor loadings and adequate discriminant validity, supporting acceptable parameter estimation and statistical performance [38].

The findings indicate that CIED patients’ intention to use RM services is significantly associated with TAM constructs. PE, PEOU, and PU all positively influenced intention to use, with PE showing the strongest effect. The core propositions of TAM were fully supported, reinforcing its applicability to health technology adoption and aligning with previous research on telemedicine, electronic health records, and mobile health applications [10].

PE is positively associated with PEOU, PU, and intention to use. A prior study based on TAM exploring the use of digital health technologies observed that one of the largest total upstream effects on associations with intention of use was PE [39]. Approximately 70% of participants perceived RM services as effective in facilitating early detection of abnormalities and in providing timely support for therapeutic adjustments when needed. These findings are consistent with evidence from a German study examining patient perspectives on smartphone-based RM services for CIEDs [40]. Patients perceived the service as advantageous due to faster responses to device-related issues, arrhythmias, and ICD shocks, as well as increased reassurance and the ability to evaluate symptoms without the need for in-person visits. These perceptions reflect the effectiveness and usefulness of the service. The RM system examined in this study employs a mode of use that is substantially less complex than smartphone-based systems; consequently, it is understandable that PE is closely related to PEOU. The interaction between PE and PU represents a meaningful mechanism in e-health service adoption. When patients recognize how the effectiveness of e-health technologies influences their healthcare outcomes, this recognition subsequently enhances their perceived usefulness of the services, thereby strengthening their intention to use them [41,42,43].

With regard to PEOU, the results indicate that it positively contributed to both PU and intention to use. Less than 30% of the CIED users in the study reported negative perceptions of the ease of use of the RM service. These findings suggest that most respondents did not perceive the mode of use of the technology as complex or difficult. PEOU is recognized as one of the essential determinants of patients’ widespread adoption of healthcare technologies and there are empirical evidences supporting its critical role in shaping adoption-related outcomes for healthcare technologies. Park et al. demonstrated that PEOU positively influences both PU and intention to use in the context of health applications [27], similarly, Kim et al. found that PEOU significantly contributed PU, which in turn drives intention to use digital health technologies [39], highlighting that users are more likely to recognize the utility of a system when it is easy to use. These findings reinforce the theoretical predictions of the TAM, indicating that designing health technologies with intuitive, user-friendly interfaces not only enhances perceived value but also strengthens adoption intentions.

PU is directly associated with intention to use; PU was rated positively by nearly 70% of participants, reflecting its potential to enhance early and effective detection of cardiac conditions, improve the quality of treatment and care for underlying heart disease, and support timely updates on both clinical and technical aspects of device management. PU has been consistently identified as a critical contributor to intention to use healthcare technologies. A review of over 140 TAM-based studies showed that PU is strongly associated behavioral intention across diverse technologies, including telemedicine, electronic health records, and mobile health applications, and often mediates the effects of other constructs such as perceived effectiveness [10]. PU exerts a robust positive association on intention to use digital health technologies among people with disabilities, surpassing perceived ease of use, and mediates the influence of usability-related factors on adoption [39]. Together, these studies underscore that enhancing the perceived utility of health technologies is essential to fostering adoption.

Time constraints, travel difficulties, and lack of accompaniment can impede patients from attending six-month CIED follow-ups, and delay or missed follow up may lead them to feel concerned about their cardiac well-being [15,27,28,29,30], potentially enhancing the intention to use RM. However, in this study, PB and PT did not moderate the intention to use RM. This may be due to Taiwan’s National Health Insurance (NHI) system, which offers high accessibility, broad coverage, and relatively short waiting times [44,45], thereby reducing the impact of PB and PT. Another possible explanation is that most participants in this study had conventional pacemakers rather than CRT or ICD devices. Pacemaker recipients generally have a lower risk of heart failure progression and life-threatening ventricular arrhythmias than CRT or ICD recipients [46], as a result, they may perceive less health threat and experience lower levels of disease-related anxiety, which may explain why PT was not significantly associated with the intention to use remote monitoring. As a result, these factors did not significantly influence the relationship between intention to use and the TAM constructs, as hypothesized in this study.

Economic considerations are often assumed to play a decisive role in the adoption of CIED-related technologies [16,47]. The results of this study indicate that, when examined as a single independent variable, economic considerations were significantly and positively associated with intention to use. From a consumer perspective, financial factors are critical in patients’ decisions to adopt healthcare technologies. Even when a technology is considered essential for managing clinical conditions, patients may perceive associated service fees as exceeding their budget, particularly when benefits are not immediately observable [16]. High out-of-pocket costs can further reduce perceived value and discourage adoption [48]. From a behavioral economics standpoint, individuals are more likely to accept pricing schemes they perceive as fair and aligned with their needs. When multiple options are available, patients tend to evaluate and select services based on personal financial constraints and expected value [34]. This highlights the importance of flexible pricing strategies that accommodate diverse preferences. A severity-adjusted pricing model may improve perceived fairness and affordability, thereby increasing acceptance of RM services. Additionally, offering varied pricing schemes—such as tiered plans, subscriptions, or pay-per-use options—can address differences in price sensitivity [34]. Providing such flexibility enables patients to select options that best match their perceived value and budget constraints, ultimately facilitating greater adoption.

The binomial logistic regression analysis in the present study indicated that intention to use RM significantly influences actual usage, which is consistent with established behavioral theories. The results suggest that fostering patients’ intention to use RM may be an important step toward increasing real-world adoption of remote monitoring technologies. In our study, only 12 of 104 respondents (11.53%) were using the RM service, despite more than half expressing positive intention. This aligns with prior research in rheumatology patients, which found that while 68.4% of patients believed in the benefits of mobile health apps, only 4.1% actively used the service [49]. In our model, the explanatory power of the model was modest (Nagelkerke R^2^ = 0.172), indicating that factors beyond intention also influence RM adoption, reflecting the complex nature of technology adoption.

We further investigated the influence of socio-demographic factors, as in the context of cardiovascular disease, socio-demographic differences in individuals’ perceptions of control over disease causation and preventable death have been documented [50,51,52]. Our study revealed that younger, college-educated, and employed patients were more likely to perceive time and distance as PB to in-person CIED follow-up and showed greater concern about delayed detection as PT, contributing to stronger intentions to use remote monitoring services. Patients with higher education showed greater PU of the service, while employed patients reported higher PE of RM.

Variables such as age, educational attainment, and income have been repeatedly shown to be associated with individuals’ overall subjective sense of control over disease and mortality in cardiovascular disease [50,51,52]. As mentioned, our younger patients showed a higher willingness to use RM than older patients. Whether advanced age itself affects the use of RM remains an open question and it is multifactorial [43]. An app-based RM study demonstrated that ongoing contact was associated with improved patient adherence to the service and increased feelings of reassurance in a smartphone-based RM service [53]. Subjective norms and social influence rather than age significantly affect the adoption of and adherence to RM technology [50,51]. These findings suggest that age alone is not a determining factor, and that other variables may influence the intention to use such services [43]. Our results demonstrated a positive correlation between the level of education and the constructs examined in this study, as well as with intention to use RM services. Consistent with previous research, patients with higher levels of education tend to be more proactive in managing cardiovascular conditions, exhibit greater health literacy, and are more likely to adopt strategies that lead to improved health outcomes [50,51]. Both employed and higher-income individuals perceived significantly greater barriers to in-clinic follow-up and higher levels of threat from their cardiac condition, this finding is consistent with previous studies [54,55]. They tended to rate the system as more effective and useful, resulting in a stronger intention to use the service. As mentioned previously, patients tend to evaluate and adopt services based on personal financial conditions and expected value [34]. Among this group of patients, work and travel constraints likely hinder clinic visits [54,55], consequently making RM services more appealing and increasing their willingness to adopt them. Telehealth technologies, such as RM, enable remote clinical care, reducing travel time and work disruptions while overcoming geographic barriers. Their accessibility and efficiency enhance patient convenience and support improved long-term care management [40], fulfilling the need and expectation in this group of patients. Additionally, these patients typically have greater resources for disease management, which may further facilitate adoption of the service, compared with retired or unemployed patients who may face limited access due to lower income and fewer resources, this reflects the delicate issue of the economic value of time and treatment affordability [54].

## 6. Limitations and Future Directions

Despite the meaningful findings of this study, several limitations should be acknowledged when interpreting the results. First, the relatively modest sample size remains a limitation and may restrict the generalizability of the study findings. Although the measurement model demonstrated strong psychometric properties, with satisfactory standardized factor loadings and adequate discriminant validity, and the binary logistic regression model demonstrated acceptable fit and calibration, the relatively small number of RM users may have reduced the precision of the estimated effect size, therefore, the findings should be interpreted cautiously. The model explained only 17% of the variance in actual RM use, suggesting that additional factors not measured in this study may contribute to RM adoption. Future studies with larger and more diverse populations are therefore recommended to enhance external validity, incorporating broader individual, technological, and organizational determinants of actual RM use. Second, this study was conducted within the context of Taiwan’s NHI system and healthcare environment, which may limit the applicability of the findings to other countries with different institutional and policy frameworks. Policymakers should consider the specific context of their own country or institution when applying these findings. Furthermore, given the cross-sectional design of this study, the identified relationships should be interpreted as associations rather than causal effects. As RM technologies continue to evolve, future longitudinal studies are warranted to validate the directionality and causality of the observed relationships. Such approaches would help reduce potential bias, strengthen the robustness of the findings, and provide a more comprehensive understanding of the dynamic relationships among the variables examined. Additionally, multiple subgroup analyses examining associations between sociodemographic factors and study outcomes may have increased the risk of Type I error. As no formal adjustment for multiple testing was applied, these findings should be interpreted with caution and confirmed in future research. Finally, the associations between sociodemographic factors and PB and PT warrant further investigation within the framework of the HBM, as the current analysis is constrained by the research model applied in this study.

## 7. Conclusions

This study examined the determinants of patients’ intention to use RM services for CIEDs in Taiwan by integrating the TAM with extended constructs. The findings support the applicability of the TAM framework in explaining health technology adoption in the CIED context. However, a notable gap between behavioral intention and actual use was identified. Economic considerations were significantly associated with patients’ intention to utilize RM services. Sociodemographic differences further influenced adoption intentions, with younger, more educated, and employed patients reporting higher perceived effectiveness and usefulness of RM services. Overall, these findings suggest that promoting RM adoption requires not only user-friendly technologies but also targeted strategies that consider patient characteristics, including educational interventions emphasizing clinical effectiveness and practical benefits. In addition, flexible pricing strategies and supportive reimbursement policies for socioeconomically disadvantaged groups may help bridge the gap between intention and sustained use of RM services.

## Figures and Tables

**Table 1:** Socio-demographic characteristics of the participants.

Socio-Demographic Characteristics		N (%)
Age	31~44 y/o	6 (5.8)
	45~64 y/o	29 (27.8)
	65~80 y/o	42 (40.4)
	Older than 80 y/o	27 (26)
Gender	Male	57 (54.8)
	Female	47 (45.2)
Education level	Primary school	30 (28.8)
	High school	35 (33.7)
	College/University	33 (31.7)
	Graduate School	6 (5.8)
Living status	Living alone	10 (9.6)
	Living with family	94 (90.4)
Income	<343,080 NTD	51 (49)
	343,080~590,000	24 (23.1)
	590,001~1,330,000	22 (21.2)
	>1,330,001	7 (6.7)
Profession	Agriculture, forestry, fishery and animal husbandry	13 (12.5)
	Legal, Business, Finance and Insurance	4 (3.8)
	Manufacturing/Construction	11 (10.6)
	Technology	7 (6.7)
	Medical/healthcare	3 (2.9)
	Accommodation and catering services	6 (5.8)
	Public Service (Military, Civil Servants, and Teachers)	14 (13.5)
	Freelance/Housekeeping	46 (44.2)
Retired	Yes	68 (65.4)
	No	36 (34.6)
Chronic diseases	No other chronic diseases	15 (14.4)
	One chronic disease	63 (60.6)
	Two or more chronic diseases	26 (25)
Diagnosis for a cardiac implantable device	Sick sinus syndrome/atrioventricular block	84 (80.8)
	Heart failure	10 (9.6)
	Ventricular tachycardia	10 (9.6)
Actual use of Remote monitoring	No	92 (88.5)
	Yes	12 (11.5)
RM service adoption by device	Pacemaker	6 (7.1)
	Cardiac resynchronization therapy (CRT)	3 (30)
	Implantable cardioverter-defibrillator (ICD)	3 (30)

**Table 2:** Factor loading, Reliability, AVE and VIF.

		Factor Loading	Cronbach’s α	CR	AVE	VIF
Perceived Barrier	PB1	0.81	0.903	0.899	0.66	1.287
	PB2	0.898				
	PB3	0.896				
	PB4	0.798				
	PB5	0.616				
Perceived Threat	PT1	0.788	0.841	0.816	0.537	1.233
	PT2	0.881				
	PT3	0.896				
	PT4	0.891				
	PT5	0.887				
Perceived Effectiveness	PE1	0.917	0.956	0.962	0.885	4.725
	PE2	0.969				
	PE3	0.937				
Perceived Ease Of Use	PEOU1	0.875	0.955	0.956	0.846	1.25
	PEOU2	0.908				
	PEOU3	0.972				
	PEOU4	0.915				
Perceived Usefulness	PU1	0.965	0.973	0.963	0.852	4.513
	PU2	0.978				
	PU3	0.86				
	PU4	0.974				
	PU5	0.874				
	PU6	0.884				
Economic considerations	E1	0.426	0.753	0.77	0.553	1.214
	E2	0.733				
	E3	0.795				
Intention to use	I1	0.902	0.965	0.967	0.906	4.133
	I2	0.979				
	I3	0.973				

**Table 3:** The heterotrait–monotrait ratio (HTMT).

	Mean	SD	PB	PT	PE	PEOU	PU	I
PB	15.962	4.982	0.812					
PT	20.625	3.782	0.152	0.732				
PE	11.385	2.174	0.079	0.475	0.940			
PEOU	16.462	3.921	0.069	0.258	0.609	0.919		
PU	22.308	4.345	0.083	0.399	0.904	0.591	0.923	
I	10.346	2.042	0.245	0.294	0.571	0.564	0.554	0.951

Note: Diagonals represents the square root of average variance extracts. SD = standard deviation. PB = Perceived Barrier; PT = Perceived Threat; PE = Perceived Effectiveness; PEOU = Perceived Ease of Use; PU = Perceived Usefulness; Intention to Use = I.

**Table 4:** The effects of sociodemographic variables analyzed by ANOVA.

		PB	PT	PE	PU	I
Age		22.00 ± 1.06	22.17 ± 0.87			12 ± 0
		16.66 ± 0.93	22.17 ± 0.58			10.86 ± 0.41
		15.74 ± 0.61	20.10 ± 0.55			10.24 ± 0.26
		5.96 ± 0.49	19.44 ± 0.87			9.59 ± 0.43
	F (df)	4.71 ** (3, 100)	3.30 * (3, 100)			3.42 * (3, 100)
	η^2^p	0.124	0.09			0.093
	Post Hoc Tukey	1 > 3 > 4	2 > 4			1 > 3
Education		13.87 ± 0.99	19.30 ± 0.84	10.90 ± 0.45	21.90 ± 0.93	9.50 ± 0.31
		17.20 ± 0.65	20.57 ± 0.43	10.80 ± 0.35	20.69 ± 0.60	9.91 ± 0.38
		15.64 ± 0.88	20.97 ± 0.59	12.06 ± 0.30	23.82 ± 0.69	11.24 ± 0.28
		21.00 ± 1.13	25.67 ± 1.38	13.50 ± 0.67	25.50 ± 1.20	12.16 ± 0.65
	F (df)	5.14 ** (3, 100)	5.50 ** (3, 100)	4.77 ** (3, 100)	4.55 ** (3, 100)	6.98 *** (3, 100)
	η^2^p	0.133	0.142	0.125	0.12	0.173
	Post Hoc Tukey	2 > 1, 3 > 1	4 > 3, 4 > 2, 4 > 1	4 > 1, 4 > 2	4 > 2, 3 > 2	4 > 2, 4 > 1,
						3 > 2, 3 > 1
Professional background						10 ± 0.40
						11 ± 0.50
						10.36 ± 0.43
						12 ± 0.65
						12 ± 0
						11.33 ± 0.49
						11.28 ± 0.46
						9.54 ± 0.34
	F (df)					3.21 ** (7, 96)
	η^2^p					0.19
	Post Hoc Tukey					4 > 8
Retired	Yes	14.76 ± 0.29	20.06 ± 0.46	11.04 ± 0.28		9.97 ± 0.24
	No	18.22 ± 0.72	21.69 ± 0.58	12.03 ± 0.29		11.06 ± 0.34
	F (df)	12.62 **(1, 102)	4.54 * (1, 102)	5.01 * (1, 102)		7.03 ** (1, 102)
	η^2^p	0.11	0.043	0.047		0.065
		No > yes	No > yes	No > yes		No > yes
Annual income		15.47 ± 0.65		10.98 ± 0.33		9.67 ± 0.270
		14.25 ± 0.98		10.83 ± 0.45		10.29 ± 0.45
		18.55 ± 1.05		12.36 ± 0.23		11.41 ± 0.32
		17.29 ± 2.11		13.14 ± 0.55		12.14 ± 0.55
	F (df)	3.48 * (3, 100)		4.54 ** (3, 100)		6.61 *** (3, 100)
	η^2^p	0.095		0.12		0.165
	Post Hoc Tukey	3 > 2		3 > 2		4 > 1, 3 > 1

Note: Age: (1) 31–44 years, (2) 45–64 years, (3) 65–80 years, (4) >80 years. Education level: (1) Primary school, (2) High school, (3) College/University, (4) Graduate school. Profession: (1) Agriculture, forestry, fishery, and animal husbandry, (2) Legal, business, finance, and insurance, (3) Manufacturing/Construction, (4) Technology, (5) Medical/Healthcare, (6) Accommodation and catering services, (7) Public service (military, civil servants, and teachers), (8) Freelance/Housekeeping. Income: (1) <343,080 NTD, (2) 343,080–590,000 NTD, (3) 590,001–1,330,000 NTD, (4) >1,330,001 NTD. PEOU, gender, living status, chronic disease, and diagnosis of a cardiac implantable device are not listed as they showed no significant differences between groups and constructs. * *p* < 0.05, ** *p* < 0.01, *** *p* < 0.001.

## Data Availability

All data used for this study are available from the corresponding author upon reasonable request.
